# A randomized, open-label, two-way crossover clinical trial to evaluate the food effect on pharmacokinetics and safety of DHP107 in patients with advanced solid tumors: the FEEL study

**DOI:** 10.1007/s00280-026-04887-9

**Published:** 2026-05-30

**Authors:** Erika Hitre, István Láng, Dénes Páll

**Affiliations:** 1 Department of Chemotherapy, National Institute of Oncology, Budapest, Hungary; 2 Clinexpert-Research, Budapest, Hungary; 3 Department of Medical Clinical Pharmacology, University of Debrecen, Debrecen, Hungary

**Keywords:** Food effect, DHP107, Oral paclitaxel, Pharmacokinetics, Safety, Efficacy

## Abstract

**Purpose:**

The phase 1, randomized, open-label, two-way crossover FEEL study assessed the effect of food on the pharmacokinetics of DHP107, an oral paclitaxel with a novel lipid formulation designed to be systemically absorbed without Cremophor EL, in patients with advanced solid tumors.

**Methods:**

Patients were randomized 1:1 to DHP107 (200 mg/m^2^ orally twice daily on Days 1, 8, 15 every 28 days for two cycles) treatment sequences: fasted–fed (first DHP107 dose administered after overnight fast; *n* = 13) and fed–fasted (first DHP107 dose administered after a high-fat meal; *n* = 12). The primary objective was to evaluate the effect of food on DHP107 pharmacokinetics. The secondary objective was DHP107 safety; efficacy was also assessed (both in all patients regardless of dosing sequence).

**Results:**

Mean systemic exposures were similar for paclitaxel and its metabolites under fed and fasted conditions. Geometric mean ratios (fed:fasted) for paclitaxel were: maximum concentration (C_max_), 101.00% (95% CI 91.93–110.95%); area under the curve (AUC_0–72_), 102.92% (95% CI 95.04–111.45%); and AUC_0–inf_ 104.76% (95% CI 96.44–113.80%), meeting criteria for no food effect. Common adverse events across the study period included diarrhea (fasted–fed 61.5%; fed–fasted 41.7%), vomiting (fasted–fed 30.8%; fed–fasted 8.3%), and neutropenia (fasted–fed 23.1%; fed–fasted 16.7%). Two fed–fasted patients (16.7%) had partial responses and six had stable disease (50.0%); seven fasted–fed patients had stable disease (53.8%).

**Conclusion:**

This study suggests that the pharmacokinetics of DHP107 are unaffected by food and DHP107 can be taken with or without food.

**Clinical trial registration:**

ClinicalTrials.gov ID: NCT04675528; 107CS-7.

**Supplementary Information:**

The online version contains supplementary material available at 10.1007/s00280-026-04887-9.

## Introduction

Paclitaxel is an antimitotic agent with proven efficacy in patients with a range of solid tumors [1, 2]. Adverse events (AEs) associated with its use, specifically peripheral neuropathy, hypersensitivity reactions, and the bone marrow suppression that results from prolonged therapy [3, 4], can limit its use. In addition, the poor water solubility of paclitaxel means that a vehicle such as Cremophor EL (BASF Corporation, Ludwigshafen, Germany) is required to aid intravenous (IV) administration. This formulation has, however, been associated with hypersensitivity reactions [5] that necessitate premedication with corticosteroids and antihistamines, as well as prolonged infusion times [6]. Consequently, much research effort has been invested in the development of improved delivery systems for this agent [7].

One alternative to IV paclitaxel is DHP107, an oral paclitaxel created using the DaeHwa-Lipid bAsed Self-Emulsifying Drug delivery system (DH-LASED), a novel lipid formulation that is systemically absorbed without the need for Cremophor EL [8]. Upon hydration in the gastrointestinal tract, DHP107 forms micelles by mixing with bile acids; the formulation adheres to the mucosal lining and microvilli of the small intestine, prolonging its retention time. This mucoadhesion is key to overcoming the low bioavailability of paclitaxel [9]. The phase 3 DREAM study (NCT0183973) in patients with advanced, metastatic, or locally recurrent gastric cancer confirmed that DHP107 was noninferior to IV paclitaxel, with comparable efficacy and safety parameters and no hypersensitivity reactions [10]. Another oral formulation of paclitaxel is Oraxol, a combination of paclitaxel and the P-glycoprotein inhibitor encequidar [11]. Efflux of paclitaxel by glycoprotein in the gastrointestinal tract results in its low bioavailability when administered orally. In a phase 3 study of combined paclitaxel + encequidar versus IV paclitaxel (NCT02594371), neuropathy was less frequent and severe with the oral formulation, although neutropenic serious infections were increased [12].

Determination of a food effect is an important consideration for orally administered agents such as DHP107. Food–drug interactions can have a significant impact on the safety and efficacy of a drug, potentially increasing or lowering systemic exposure to the drug, as well as increasing or reducing adverse effects such as nausea and vomiting [13]. Food effect bioavailability studies are therefore designed to determine the effects of food on the rate and extent of drug absorption when the drug is administered shortly after a meal (fed conditions) versus administered before a meal (fasted conditions).

Patients in previous studies took DHP107 at different timepoints relative to food: patients in the phase 1 study took DHP107 2 hours after a light breakfast followed by a 2-hour fast [14], DHP107 was taken approximately 1 h after breakfast and dinner in the phase 2 OPTIMAL study [15], and DHP107 was taken within 1 h of meals in the phase 3 DREAM study [10]. In the phase 1 study, which included a pharmacokinetics substudy, AUC_inf_, indicating the drug absorption according to increasing doses of DHP107, increased in a proportional manner to the dose up to 300 mg/m^2^. At higher levels, however, there were no further proportional increases depending on the increased dose, as the difference between species became greater. The maximal plasma concentration (C_max_) was 131 ng/mL in the initial dose group (60 mg/m^2^), higher than the 42 ng/mL previously reported to be an effective therapeutic dose [14]. C_max_ increased to 446 ng/mL at the 550 mg/m^2^ dose. The half-life varied between 12 and 24 h, and T_max__,_ the time to reach the maximal plasma level, varied between 2.4 and 4.7 h. Furthermore, in clinical trial simulations, the most frequently predicted maximum tolerated dose of DHP107 was 480 mg/m^2^ [16].

The phase 1 FEEL study was undertaken to determine the effect of food on the pharmacokinetics (PK) of DHP107 under fed and fasted conditions in patients with advanced solid tumors. Evaluation of the safety of DHP107 in patients with advanced solid tumors was a secondary objective of the study.

## Methods

### Study design

**Fig. 1:**
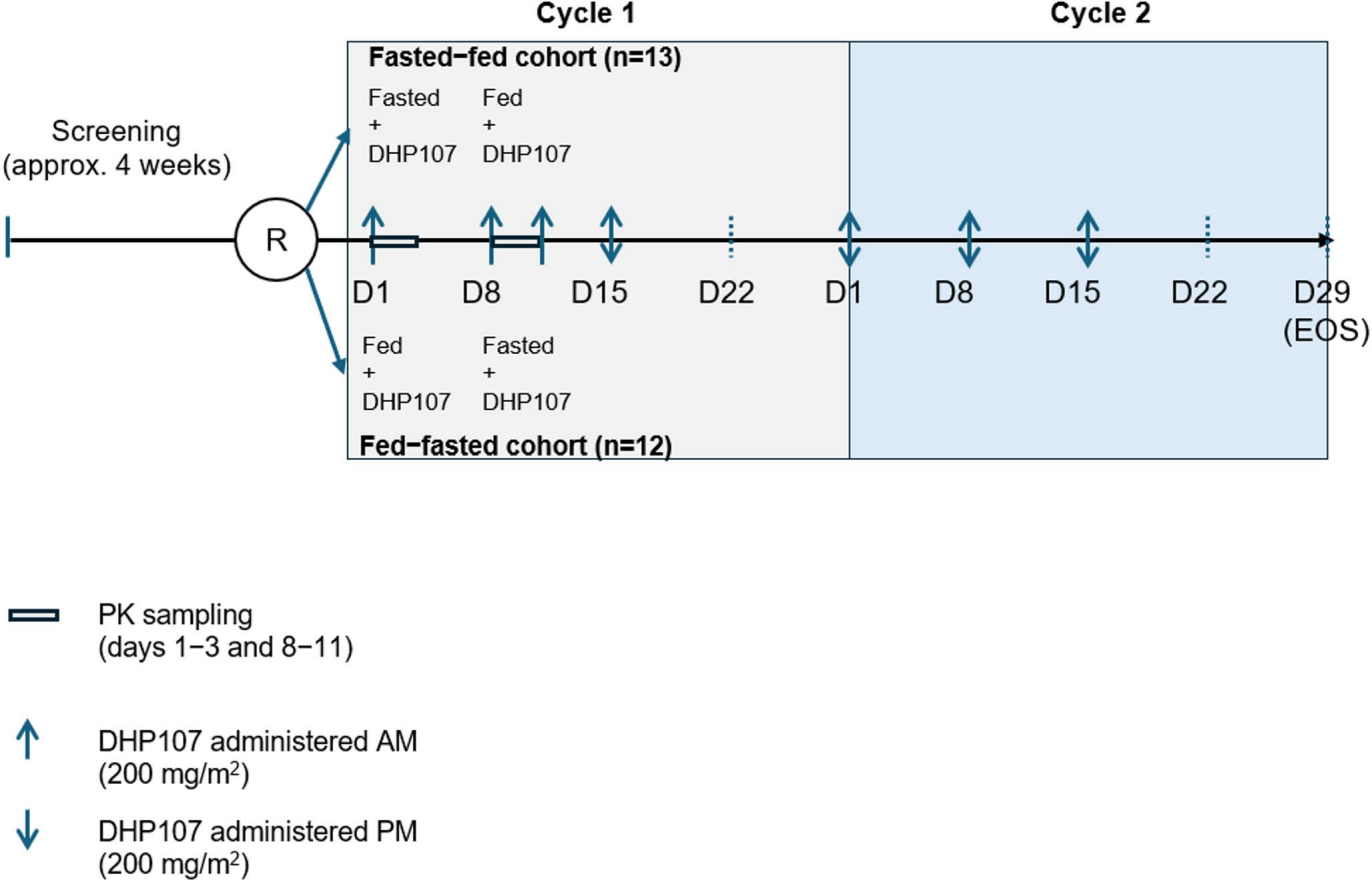
Study design. D day, entry at investigator’s discretion, EOS end-of-study visit, PK pharmacokinetic, R randomized

This phase 1, randomized, open-label study (NCT04675528) had a two-way crossover design consisting of two sequences: Sequence 1 (fasted–fed treatment) and Sequence 2 (fed–fasted treatment). The study was conducted at five centers in Hungary in patients with advanced solid tumors. The study design is summarized in Fig. 1.

Patients were screened for eligibility for approximately 28 days, then randomly assigned 1:1 to one of the two treatment sequences before administration of the first dose of study treatment (DHP107 200 mg/m^2^ orally). In Sequence 1 (fasted–fed), patients received a DHP107 with 100 mL of water after an overnight fast of at least 10 h (fasted state). No food was allowed for at least 4 h post-dose. The evening dose was omitted on this day to allow a full 24-hour blood sampling period that would include accurate observation of the elimination phase. On day 8, patients received a high-fat, high-calorie meal (Supplementary methods), followed by DHP107 at the end of the meal with 100 mL of water (within 30 min of the start of the meal; fed state). No food was allowed for at least 4 h post-dose. The evening dose was administered on day 11, after completion of the PK sampling. In Sequence 2 (fed–fasted), patients received DHP107 in the fed state on day 1 and in the fasted state on day 8. As in Sequence 1, the evening dose was omitted on day 1 and the evening dose on day 8 was administered on day 11, after the last PK sampling.

For the remainder of the study, patients received DHP107 twice daily on Days 1, 8, and 15 of a 28-day cycle for two cycles; no patients received study drug for more than two cycles. No premedication was allowed during Cycle 1; however, premedication for preventive purposes could be administered at the investigator’s discretion in Cycle 2. The scheduled end-of-study (EOS) visit was Day 29 of Cycle 2. Patients visited the study center on Days 0, 7, and 15 of Cycle 1 and Days 1 and 29 of Cycle 2; patients were hospitalized overnight on Days 0 and 7 to maintain the overnight fast. Patients were not required to visit the study center on Days 8 and 15 of Cycle 2 unless requested to by the investigator. Those who did not attend were assessed by phone for AEs, concomitant medications, and drug compliance. If a patient was withdrawn from the study before the end of Cycle 2, an EOS visit was conducted within 28 days (+ 7 days) after the last administration of DHP107 or immediately before initiation of any other treatment. At this visit, safety evaluations were performed (physical examination and collection of AEs, toxicities, laboratory evaluations, pregnancy test, electrocardiogram), as well as tumor evaluation and Eastern Cooperative Oncology Group (ECOG) performance status (PS) evaluation.

The study was conducted in accordance with principles of the Declaration of Helsinki and Good Clinical Practice guidelines. The protocol, informed consent forms, and other related documents were reviewed and approved by the competent authority and a central ethics committee.

### Patients

Eligible patients were 18 years of age or older, had histologically or cytologically confirmed advanced solid tumors that were measurable according to Response Evaluation Criteria in Solid Tumours (RECIST) version 1.1, an ECOG PS of 0, 1, or 2, and a life expectancy of ≥ 12 weeks. All patients were required to provide informed consent before inclusion in the study. Patients had to have adequate organ function, defined as: absolute neutrophil count ≥ 1.5 × 10^9^/L; hemoglobin ≥ 9 g/dL; platelet ≥ 100 × 10^9^/L; serum creatinine ≤ 1.5 × institutional upper limit of normal (ULN; patients with creatinine clearance or estimated glomerular filtration rate of ≥ 60 mL/min were eligible); serum total bilirubin within normal limits, or ≤ 1.5 × ULN (although higher levels due to Gilbert’s syndrome were allowed); and serum alanine aminotransferase and aspartate aminotransferase ≤ 2.5 × ULN, or ≤ 5 × ULN for patients with hepatic metastases. Patients had to be able to take oral medication. Exclusion criteria included a history of severe hypersensitive reaction to the active ingredients or excipients of DHP107, surgical or medical conditions what would affect drug absorption, grade ≥ 2 neuropathy at study entry, symptomatic or unstable central nervous system metastases, or inability to take the high-fat meal required for the food study (Supplementary methods). A complete list of inclusion and exclusion criteria is shown in Supplementary Table S1.

### Endpoints and assessments

The primary endpoints were the ratio of geometric means of the following PK parameters following DHP107 administration: maximum observed concentration (C_max_); area under the plasma concentration–time curve over the last 24-hour interval (AUC_0−24_); and AUC from time zero to infinity (AUC_inf_); plus the median difference of the following PK parameters following DHP107 administration: time of maximum observed concentration (T_max_); half-life (t_1/2_) of DHP107; apparent plasma concentration (CL/F); and apparent volume of distribution based on area (V_Z_/F). Secondary endpoints included: AEs, treatment-emergent AEs (TEAEs), and serious AEs (SAEs) according to National Cancer Institute Common Terminology Criteria for Adverse Events (NCI-CTCAE) v5.0; abnormal laboratory tests (hematology and biochemistry); physical findings; changes in 12-lead electrocardiogram (ECG) tests (shift from normal to abnormal); and changes in ECOG PS, weight, and vital signs. Response was assessed according to RECIST version 1.1 at the screening and at the end of the study. This efficacy analysis was secondary and was used only in order to judge if the patient qualified to continue to the extended phase of the study.

### Pharmacokinetics analysis

Blood samples (4 mL) were collected for PK assessments on Days 1 and 8 of Cycle 1 at pre-dose and 1, 2, 3, 4, 6, 8, 12, 24, 36, 48, and 72 h post-dose. All PK samples were analyzed by a sponsor-designated third party using an appropriate bioanalytical method. Samples were further analyzed to explore the presence of circulating metabolites 6α-hydroxypaclitaxel and 3′-p-hydroxypaclitaxel following DHP107 administration using a liquid chromatography tandem mass spectrometry (LC–MS/MS) method with electrospray ionization in the positive mode. The lower limit of quantification was 0.3 ng/mL and the upper limit of quantification (without dilution) was 300 ng/mL. Results of the validation of the LC–MS/MS method are shown in the Supplementary Methods (Table S2).

Pharmacokinetic parameters for paclitaxel and 3′-hydroxypaclitaxel in plasma were compared under fasted and fed conditions. The analysis of variance (ANOVA) method was used to perform logarithmic transformation of key parameters, and a mixed effect model was used, with fasted/fed treatment, sequence, and period as fixed effects and patients as random effects. The geometric mean ratio and 90% confidence interval of the parameters under fasted or fed conditions were calculated.

### Statistical methods

The primary analysis was designed to compare the fed condition with the fasted condition with respect to the PK parameters of DHP107. Individual patient C_max_, AUC_0−24_, AUC_0−72_, AUC_inf_, T_max_, t_1/2_, CL/F, and V_Z_/F values were derived using noncompartmental analysis of paclitaxel concentration–time data. Absence of food effect on bioavailability was when the 90% CI for the ratio of population geometric means between fed and fasted treatments, based on log-transformed data, was within the equivalence limits of 80−125% for AUC and C_max_ [17]. Safety events were collected and evaluated to guarantee the safety of participating patients but not to identify significant differences between the two modes of dosing (fasted and fed). No formal comparison between the fed–fasting and fasting–fed groups was conducted.

Counts and percentages were provided for categorical variables, and means, standard deviations, medians, and ranges were provided for continuous variables.

## Results

### Patients

Between May 18 2021 and August 15 2022, 32 patients provided informed consent and underwent screening, seven of whom were screen failures (failed to meet inclusion criteria, *n* = 5; withdrew from the study, *n* = 2). This study therefore enrolled 25 patients, 12 randomized to the fed–fasted sequence and 13 randomized to the fasted–fed sequence. One patient discontinued treatment because of an SAE (dehydration). The pharmacokinetic population, which included patients who completed all required study visits, comprised 21 patients (84.0%), 11 (84.6%) in the fasted−fed group and 10 (83.3%) in the fed−fasted group. All 25 patients were included in the safety population. 

Patient characteristics are summarized in Table 1. All patients had stage IV/metastatic disease, with the exception of two in the fasted −fed group and one in the fed−fasted group who had locally advanced disease.

**Table 1:** Patient characteristics at baseline

	Fasted–fed^a^ (*n* = 13)	Fed–fasted^b^ (*n* = 12)	All patients (*n* = 25)
Median age, y (range)	66 (39–72)	57.5 (36–78)	65 (36–78)
Sex, n (%)			
Female	9 (69.2)	10 (83.3)	19 (76.0)
Male	4 (30.8)	2 (16.7)	6 (24.0)
Tumor type, n (%)			
Breast	6 (46.2)	4 (33.3)	10 (40.0)
Lung	4 (30.8)	3 (25.0)	7 (28.0)
Parotid gland cancer	1 (7.7)	2 (16.7)	3 (12.0)
Head and neck	0	1 (8.3)	1 (4.0)
Adenoid cystic	0	1 (8.3)	1 (4.0)
Planocellular	1 (7.7)	0	1 (4.0)
Orbit	1 (7.7)	0	1 (4.0)
Synovial sarcoma	0	1 (8.3)	1 (4.0)
Median time since metastatic diagnosis, y (range)	2.0 (0.10–6.29)	1.5 (0.01–9.30)	1.8 (0.01–9.30)
Stage, n (%)			
IIIA	0	1 (8.3)	1 (4.0)
IIIB	0	0	0
IIIC	2 (15.4)	1 (8.3)	3 (12.0)
IV	11 (84.6)	11 (91.7)	22 (88.0)
Prior surgery, n (%)	9 (69.2)	5 (41.7)	14 (56.0)
Prior chemotherapy, n (%)	10 (76.9)	10 (83.3)	20 (80.0)
Prior radiotherapy, n (%)	7 (53.8)	6 (50.0)	13 (52.0)

^a^Received DHP107 in fasted state on Day 1 and fed state on Day 8. Received DHP107 in fed state on Day 1 and fasted state on Day 8

### Pharmacokinetics

A total of 588 plasma samples were collected from 25 patients and analyzed for paclitaxel and its metabolites. Four patients were excluded from the PK population. One patient was an outlier with below quantification levels for paclitaxel and its metabolites on Days 1 and 8. Two patients received different DHP107 doses on Days 1 and 8 (*n* = 2): both patients received a higher dose on Day 8 than on Day 1 despite their weight change being less than 10% and were therefore classed as having received an overdose. The final patient missed a dose (*n* = 1). The PK population therefore included 21 patients.

**Fig. 2:**
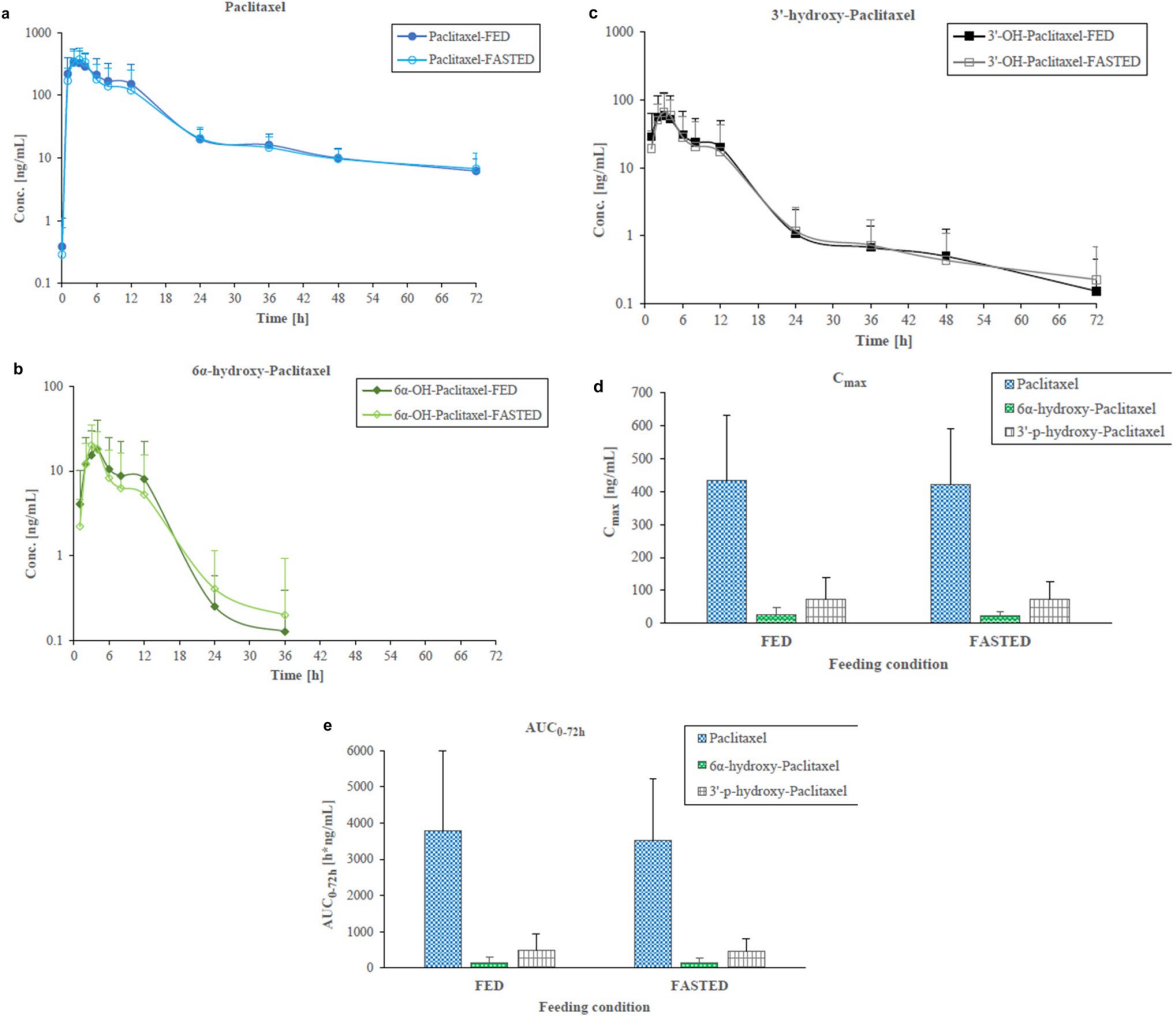
Mean plasma level curves of **a** paclitaxel and its metabolites, **b** 6-α-hydroxypaclitaxel and **c** 3’-p-hydroxypaclitaxel under fed and fasted conditions (fed–fasted and fasted–fed sequences evaluated together); **d** mean C_max_ and **e** mean AUC_0–72 h_ values of paclitaxel and its metabolites 6-α-hydroxypaclitaxel and 3’-p-hydroxypaclitaxel in patients under fasted and fed conditions

Although plasma concentrations of all analytes showed high inter-individual variability, there were no statistically significant differences in mean systemic exposures for paclitaxel and its metabolites under fed and fasted conditions (Fig. 2). A difference in the secondary concentration elevation was observed; this was slightly more pronounced in the fed state for both paclitaxel and its metabolites.

Paclitaxel PK parameters are shown for fed and fasted conditions in Table 2. Mean systemic exposures were comparable under fed and fasted conditions for paclitaxel and its metabolite 3-hydroxypaclitaxel as shown in Table 3. The reported 90% CIs for the ratio of population geometric means between fed and fasted treatments were within the equivalence limits of 80–125% for AUC and C_max_, satisfying FDA Guidance for Industry for Food-Effect Bioavailability and Fed Bioequivalence Studies regarding absence of a food effect.

**Table 2:** Total mean pharmacokinetic parameters of paclitaxel (fed–fasted and fasting–fed sequences evaluated together; *n* = 21)

Parameter	Fasted–fed^a^			Fed–fasted^b^		
	Mean (SD)	CV%	Median (range)	Mean (SD)	CV%	Median (range)
C_max_, ng/mL	423 (169)	40.0	445 (175–727)	436 (195)	44.7	403 (117–754)
AUC_0−24_, h*ng/mL	2990 (1520)	50.8	2350 (1080–7000)	3240 (2020)	62.3	2750 (841–7550)
AUC_inf_, h*ng/mL	3810 (1850)	48.6	3290 (1420–8360)	4060 (2330)	57.4	3560 (1300–8830)
T_max_, h	3.1 (1.04)	33.5	3 (1–6)	3.81 (2.52)	66.1	3 (1–12)
t_1/2_, h	28.0 (12.6)	45.0	24.5 (14.3–75.1)	32.7 (19.1)	58.4	26.3 (15.6–91.4)
CL/F, L/h/m^2^	65.7 (32.6)	49.6	60.7 (23.9–141.0)	68.3 (40.7)	59.6	57.7 (22.6–154.0)
V_Z_/F, L/m^2^	2490 (1110)	44.6	2700 (657–4930)	2930 (2050)	70.0	2600 (729–7330)

Four patients were excluded from the mean calculation for the following reasons: PK outlier (*n* = 1); overdosed resulting in different doses on Days 1 and 8 (*n* = 2), and missing the Day 8 dose (*n* = 1)

*AUC*_*0-24*_ Area under the curve from time 0 to 24 h post-dose, *AUC*_*inf*_ Area under the curve from time 0 extrapolated to infinity, *CL/F* Apparent plasma clearance, *C*_*max*_ Maximum observed concentration, *T*_*max*_ Time of maximum observed concentration, *t*_*1/2*_ half-life, *V*_*Z*_*/F* Apparent volume of distribution based on area

^a^Received DHP107 in fasted state on Day 1 and fed state on Day 8. ^b^Received DHP107 in fed state on Day 1 and fasted state on Day 8

**Table 3:** Total mean pharmacokinetic parameters of paclitaxel and its metabolites for all patients (*n* = 21)

Parameter	Geometric mean of fasted (GeoLSM)	Geometric mean of fed (GeoLSM)	Geometric mean ratio, % (Fed:Fasted)	90%CI lower limit	90%CI upper limit
Paclitaxel					
AUC_0−inf_, h*ng/mL	3030	3174	104.76	96.44	113.80
AUC_0−72_, h*ng/mL	2776	2857	102.92	95.04	111.45
C_max_, ng/mL	326	330	101.00	91.93	110.95
3′-hydroxypaclitaxel					
AUC_0−72_, h*ng/mL	270	251	92.79	82.28	104.64
C_max_, ng/mL	45.4	42.6	93.66	82.07	106.89

Four patients were not included in this analysis (one PK outlier; two who overdosed resulting in different doses on Days 1 and 8; one missing the Day 8 dose), which was done using the Phoenix WinNonlin bioavailability evaluation software (version 8.4; bioequivalence module)

The 6-α-hydroxypaclitaxel/paclitaxel ratio was very low and is not include in the above analysis (C_max_ of approximately 5% and AUC_0−72 h_ of approximately 4%)

*AUC*_*0−72*_ Area under the curve from time 0 to 72 h post-dose, *AUC*_*inf*_ Area under the curve from time 0 extrapolated to infinity, *C*_*max*_ Maximum observed concentration, *LSM* least-squares mean

### Safety

During the study period, 100 AEs were reported in 18 patients (72.0%), 10 (76.9%) in the fasted −fed group (57 events) and eight (66.7%) in the fed−fasted group (43 events). Ten SAEs were reported in six patients (24.0%; fasted −fed, *n* = 4; fed−fasted *n* = 2). A summary of AEs is shown in Supplementary Table S2.

The most common TEAEs in the overall population were diarrhea (*n* = 13; 52.0%), vomiting (*n* = 5; 20.0%), asthenia (*n* = 5; 20.0%), and alopecia (*n* = 5; 20.0%). Table 4 summarizes the most common TEAEs by grade. Overall, the number of grade 3 and 4 events was low, with eight grade 3 events in five patients and eight grade 4 events in four patients. There were no grade 5 TEAEs.

**Table 4:** Most common treatment-emergent adverse events by grade (occurring in > 10% of patients overall; safety population; *n* = 25)

Adverse event, *n* (%)	Fasted–fed^a^ (*n* = 13)				Fed–fasted^b^ (*n* = 12)			
	Grade 1	Grade 2	Grade 3	Grade 4	Grade 1	Grade 2	Grade 3	Grade 4
Diarrhea	6 (46.2)	1 (7.7)	2 (15.4)	0	4 (33.3)	1 (8.3)	1 (8.3)	0
Vomiting	2 (15.4)	2 (15.4)	0	0	1 (8.3)	1 (8.3)	0	0
Neutropenia	0	1 (7.7)	0	2 (15.4)	0	1 (8.3)	0	1 (8.3)
Asthenia	1 (7.7)	2 (15.4)	0	0	1 (8.3)	1 (8.3)	1 (8.3)	0
Alopecia	3 (23.1)	0	0	0	2 (16.7)	0	0	0
Cough	1 (7.7)	1 (7.7)	0	0	0	1 (8.3)	0	0
Decreased appetite	0	2 (15.4)	0	0	1 (8.3)	0	0	0
Dehydration	0	1 (7.7)	0	1 (7.7)	0	0	0	2 (16.7)
Nausea	0	1 (7.7)	1 (7.7)	0	1 (8.3)	1 (8.3)	0	0

Treatment-emergent adverse events were events that started after, or were present prior to, the first dose of DHP107 but increased in severity after the first dose of DHP107

^a^Received DHP107 in fasted state on Day 1 and fed state on Day 8. ^b^Received DHP107 in fed state on Day 1 and fasted state on Day 8

The most common study drug-related TEAE was diarrhea (*n* = 13); three patients had study drug-related neutropenia and three had study drug-related dehydration. Six patients had TEAEs that resulted in permanent discontinuation of study treatment. These were dehydration (*n* = 2), febrile neutropenia (*n* = 1), leukopenia (*n* = 1), neutropenia (*n* = 1), diarrhea (*n* = 1), and lung abscess (*n* = 1).

In a supplementary analysis of safety events in the fed and fasted states, there were 34 AEs during the first two weeks of treatment when these states could be clearly differentiated. Only minor differences in the number of TEAEs in the two groups were observed (Supplementary Table S4). There were three SAEs in two patients in the fed state (dehydration and neutropenia in one patient with stage IIIC lung cancer and dehydration in one patient with stage IV lung cancer). These were considered by the investigator to be possibly related to the treatment, but considering the nature of the SAEs, more likely due to the patient’s underlying disease. Twelve gastrointestinal AEs were recorded, nine of which were considered related to treatment. All nine of these treatment-related gastrointestinal events occurred in patients who received DHP107 in the fasting state.

### Efficacy

In nine patients in the fasted −fed group who had ECOG PS values at EOS, scores remained unchanged in six patients (54.5%), improved by 1 point in one patient (9.1%), and deteriorated by 1 and 2 points in one patient each (9.2%). Among nine patients with ECOG values at EOS in the fed−fasted group, scores were unchanged at the EOS in eight patients (80.0%), and deteriorated by 1 point in one patient (10.0%).

Among the 12 patients in the fed–fasted group, there were two patients with partial responses (16.7%) and six patients with stable disease (50.0%) per RECIST 1.1. Among the 13 patients in the fasted–fed group, there were seven patients with stable disease (53.8%).

## Discussion

Food can change the rate and extent of absorption of orally administered drugs. This can have considerable effects on the safety and efficacy of such agents, with the result that determination of a food effect is an important step in the development of oral anticancer agents. Furthermore, establishing that an agent does not have a food effect provides additional convenience in dosing, enabling the drug to be given before or after food. DHP107 is the new lipid-based oral semi-solid paclitaxel formulation that is solubilized by a mucoadhesive lipid drug delivery system and orally bioavailable without P-glycoprotein inhibitors. The phase1, open-label, two-way crossover, food effect FEEL study described in this report investigated the PK of DHP107 administration under fasted conditions and after a high-fat, high-calorie meal in subjects with advanced solid tumors.

FDA guidance for food effect bioavailability and fed bioequivalence studies defines the absence of food effect on bioavailability when the 90% CI for the ratio of population geometric means between fed and fasted treatments, based on log-transformed data, is within the equivalence limits of 80 −125% for AUC and C_max_ [17]. In the present study, AUC and C_max_ values for paclitaxel, 6α-hydroxy-paclitaxel, and 3′-p-hydroxy-paclitaxel after a single oral administration of DHP107 200 mg/m^2^ were unequivocally within the equivalence limits of 80 −125% in fed and fasted patients with advanced solid tumors. These data suggest that DHP107 may be taken with or without food and are in agreement with the findings of a study into the effect of food on albumin-bound paclitaxel [18].

Observed differences in secondary concentration elevations in the fed state are likely a combination of factors, such as enterohepatic recirculation and variations in gastrointestinal motility; alternatively, redistribution of circulation due to change in body position or motion can also have an impact. Paclitaxel is primarily metabolized by the liver and excreted into the bile. Once in the intestine, the drug can be reabsorbed back into the bloodstream, leading to a secondary rise or temporary plateau in plasma concentration. Irregular gastric emptying and gastrointestinal transit time can also have an impact on the absorption, causing a secondary peak. Other contributing factors may include varying pH levels, bile salt concentrations, and transporter protein expression such as P-glycoprotein. Paclitaxel is a high-affinity substrate for P-glycoprotein, an efflux transporter that pushes the drug back into the intestinal lumen. Differences in these factors along the gastrointestinal tract can lead to differential absorption at various points in time, resulting in multiple peaks in the concentration–time profile. Finally, the oral formulation itself may also play a part. Some formulations are designed to enhance solubility and absorption, e.g., lipid-based formulations like DHP107, or co-administration with P-glycoprotein inhibitors like encequidar. Interaction of these formulations with the physiological environment over time can affect absorption patterns, potentially causing secondary peaks. Assessment of these factors was beyond the scope of the present study, but may warrant further investigation in the future.

The most common AEs in the treatment groups were diarrhea and vomiting, AEs that are generally manageable with antidiarrheal and antiemetic agents. These TEAEs are likely attributable to DHP107 treatment and not connected to antiemetic premedication. Overall, the number of grade 3 and 4 events was small, with only eight grade 3 events in five patients and eight grade 4 events in four patients. No further conclusions could be drawn based on the small number of events and study design, which did not include safety analysis as a primary objective. Similarly, although the incidence of AEs in the analysis of events in the fed and fasted states appeared to be largely comparable, some differences were observed in the incidences of gastrointestinal AEs that are interesting but would require further analysis in large and longer studies.

There were no hypersensitivity reactions or neuropathy, and bone marrow suppression was also uncommon. Peripheral neuropathy is a concern not only with paclitaxel but also with some of its newer formulations, including nab-paclitaxel [19, 20]. Symptoms peak approximately 2–3 days after each dose of paclitaxel and can be severe enough to warrant treatment discontinuation in some cases [21]. The phase 3 DREAM study provided evidence that sensory neuropathy was statistically significantly lower in patients who received DHP107 versus IV paclitaxel (21.2% vs. 44.1%, respectively; *p* < 0.001) [10]. The results of the FEEL study therefore provide further evidence to suggest that DHP107 may be an effective alternative to IV paclitaxel in the treatment of patients with a range of advanced solid tumors.

Efficacy findings suggest that responses occurred in both cohorts of patients undergoing treatment with DHP107. The number of patients in the study was small, however, limiting the conclusions that can be drawn based on these findings. The fact that the observation period was limited to two treatment cycles, 56 days in total, is an additional limitation in interpretation of the efficacy data. Further studies are required to confirm the overall efficacy of DHP107 in larger numbers of patients. The study was performed in five centers, but no center-effect testing was performed as standardized procedures were followed at all centers, all of which were qualified and certified phase 1 study centers. One other limitation worthy of consideration is that patients received DHP107 monotherapy, whereas combination with another chemotherapy agent might represent a more standard approach in this setting.

A further limitation of the study is that a comparison of pharmacokinetic data for oral and IV formulations is not currently possible due to the lack of an IV paclitaxel arm. Previously reported data from a phase 3 study of IV paclitaxel in patients with ovarian cancer estimated a mean AUC after a 3-hour infusion of 7952 ng.h/mL for a 135 mg/m^2^ dose and 15,007 ng.h/mL for a 175 mg/m^2^ dose [22]. This low bioavailability means that comparative pharmacokinetics of IV paclitaxel and its oral alternatives are of interest; dedicated studies to define the pharmacokinetics of DHP107 versus IV paclitaxel therefore warrant consideration.

## Conclusions

In summary, the results of this food effect study have shown that the PK of DHP107 are unaffected by food and that DHP107 can therefore be taken before or after meals. Further studies are needed to confirm whether the suggestion of a food effect on efficacy is apparent in larger populations.

## Supplementary Information

Below is the link to the electronic supplementary material.

Supplementary Material 1

Supplementary Material 1 REVISED

## Data Availability

The data that support the findings of this study are available from Daehwa Pharmaceutical Co., Ltd., but restrictions apply to the availability of these data, which were used under license for the current study, and so are not publicly available. Data are, however, available from the authors upon reasonable request and with permission of Daehwa Pharmaceutical Co., Ltd.
