# Supplementary material for: A randomized, open-label, two-way crossover clinical trial to evaluate the food effect on pharmacokinetics and safety of DHP107 in patients with advanced solid tumors: the FEEL study
Source: Cancer Chemother Pharmacol. 2026 May 30;96(1):59. doi: 10.1007/s00280-026-04887-9 (PMC13221415; doi:10.1007/s00280-026-04887-9)
Supplement: Supplementary file 1 — Supplementary Material 1 [file 280_2026_4887_MOESM1_ESM.docx]

# A randomized, open-label, two-way crossover clinical trial to evaluate the food effect on pharmacokinetics and safety of DHP107 in patients with advanced solid tumors: The FEEL study

🖂 Erika Hitre^1*^

hitre.erika@oncol.hu

🖂 István Láng^2^

prof.lang.istvan@clinexpert.hu

🖂 Dénes Páll^3^

pall.denes@unideb.hu

Affiliations:

^1^Department of Chemotherapy, National Institute of Oncology, Budapest, Hungary. ^2^Clinexpert-Research, Budapest, Hungary.

^3^Department of Medical Clinical Pharmacology, University of Debrecen, Debrecen, Hungary

^3^Department of Medicine, University of Debrecen, Debrecen, Hungary

***Corresponding author:** Dr Erika Hitre, [hitre.erika@oncol.hu](mailto:hitre.erika@oncol.hu)

# Supplement

## Supplementary methods

The high-fat meal after which the fed cohort received their medication was as follows:

**Breakfast menu (993.5 kcal; protein, 30.73 g; carbohydrates, 35.78 g; fat, 53.45 g)**

- 2 strips of bacon: 2 x 106 kcal = 212 kcal (protein, 3.3 g; carbohydrates 0.2 g; fat 10.2 g)
- 2 rolls (kifli) (44 g/roll): 2 x 133 kcal = 266 kcal (proteins: 4.5 g; carbohydrates: 25.6 g; fat 1.1 g)
- 25 g Hungarian chorizo (téliszalámi): 133 kcal (protein: 6.25 g; carbohydrates: 0.08 g; fat 11.52 g)
- 25 g chorizo slices (szeletelt szárazkolbász): 120 kcal (protein: 5.5 g; carbohydrates: 0.25 g; fat 10.75 g)
- 2 eggs (44 g/egg): 126 kcal (protein: 11.06 g; carbohydrates: 0.64 g; fat 8.36 g)
- 14 g butter: 102 kcal (protein: 0.12 g; carbohydrates: 0.01 g; fat 11.52 g)
- 1 cup of black tea (200 ml): 2 kcal (protein: 0 g; carbohydrates: 0.6 g; fat 0 g)
- 2 teaspoons of sugar (8.4 g): 32.5 kcal (protein: 0 g; carbohydrates: 8.4 g; fat 0 g)

The meal was consumed with 100 ml water; DHP107 was administered 30 minutes after the start of the meal.

**Bioanalytical methods**

Plasma concentrations of paclitaxel, 6α-hydroxy-paclitaxel, 3’-p-hydroxy-paclitaxel were simultaneously measured using an LC–MS/MS method with electrospray ionization in the positive mode. The calibration range of the method was 0.3−300 ng/mL for all analytes in human plasma (anticoagulant: K2EDTA). Paclitaxel-d5 served as internal standard (IS).

The plasma samples were extracted by tert-butyl methyl ether and the upper organic phase was evaporated under nitrogen stream. The evaporation residue was reconstituted in methanol. After the sample clean-up procedure, the processed plasma was subjected to high performance liquid chromatography separation. The eluents consisted of acetonitrile, water, and acetic acid. The analytes and IS were quantified with multiple reaction monitoring detection at the transitions:

- Paclitaxel: 854.4→286.1 m/z;
- 6α-hydroxy-paclitaxel: 870.5→525.3 m/z;
- 3’-p-hydroxy-paclitaxel: 870.5→302.1 m/z;
- paclitaxel-d5: 859.4→291.1 m/z.

For quantification of analytes, the relationship between concentration and peak area ratios (analyte/IS) was used. Standard curve linearity and quantitation were determined by applying a recession model of weighted linear (1/concentration^2^).

All chromatographic data were collected and analyzed using Analyst 1.6.2 software. The peak area of analytes and its ratio to the area of IS were calculated automatically by the computing software. Calibration curves were constructed on the basis of response ratio of analyte to the IS versus the ratio of the concentration of analytes to the IS concentration. The weighting type was set to 1/x^2^.

The equations of the calibration curves were used to determine back-calculated concentrations for each calibrator, QC samples and the concentration of analyte in the test samples from its measured peak area ratios. The accuracy was calculated according to the following equation: accuracy [%] = (measured - nominal) / nominal concentration * 100.

The validation of the above analytical method was performed in a separate Good Laboratory Practice-compliant study at Advanced Technology Research Council (ATRC) according to SOP LAB 101 “Bioanalytical method validation”: study no.: 950.112.6045 (Validation of an LC–MS/MS method for the quantification of paclitaxel, 6α-hydroxy-paclitaxel and 3’-p-hydroxy-paclitaxel in human plasma).

The method was validated in terms of accuracy, precision, selectivity, sensitivity, linearity, recovery, matrix effect, effect of hemolyzed or lipemic plasma, carry-over and dilution effect and effect of long run. Short-term stability of the analytes in the stock and spiking solutions, processed samples, and human plasma and blood were investigated after storage at room temperature. The stability of the analytes in the stock and working solutions was also investigated after long-term storage in a freezer. The stability of paclitaxel, 6α-hydroxy-paclitaxel, and 3’-p-hydroxy-paclitaxel was demonstrated in frozen plasma after three freeze/thaw cycles and long-term storage in an ultra-freezer below -70°C. These experiments confirm that the method met the postulated acceptance criteria of validation.

The results of the validation are summarized in [Table S2](#_bookmark41) for all analytes.

**Table S1** Inclusion and exclusion criteria

| **Inclusion criteria:** |
| --- |
| 1. Patients who are ≥ 18 years of age on the date of written informed consent.  2. Patients with histologically or cytologically confirmed advanced solid tumors, including but not limited to the listed below, for which paclitaxel monotherapy has been determined an appropriate therapy at the investigator’s discretion:  - Angiosarcoma  - Bladder cancer  - Breast cancer  - Cervical cancer  - Head and neck cancer (if no difficulty with swallowing)  - Kaposi’s sarcoma  - Lung cancer  - Ovarian cancer.  3. Patients who have a life expectancy of ≥ 12 weeks.  4. Patients who are able to take oral medication.  5. Patients who have a performance status of ≤ 2 on the Eastern Cooperative Oncology Group scale.  6. Patients who have evaluable disease according to the Response Evaluation Criteria in Solid Tumors Version 1.1.  7. Patients who have adequate organ functions as indicated by the following laboratory values:  - Absolute neutrophil count ≥ 1.5 x 10^9^/L  - Platelet ≥ 100 x 10^9^/L  - Hemoglobin ≥ 9 g/dL  - Serum creatinine ≤ 1.5 x institutional ULN; however, a patient who has a CrCl or eGFR ≥60 mL/min is eligible  - Serum total bilirubin ≤1.5 x ULN (however, higher bilirubin levels due to Gilbert’s syndrome are allowed)  - Serum aspartate aminotransferase and alanine aminotransferase ≤ 2.5 x ULN (or ≤ 5 x ULN for patients with liver metastases).  8. Patients who are willing and able to comply with scheduled visits, treatment plans, laboratory tests, and procedures.  9. Patients who have voluntarily agreed to participate by giving written informed consent.  10. Women of childbearing potential who have negative pregnancy test results at the screening visit and men with female partners of childbearing potential must agree to use adequate contraception for the duration of the trial and up to 90 days after last dose of study drug. |
| **Exclusion criteria** |
| 1. Patients who have history of severe hypersensitive reaction to the active ingredient or any excipients of DHP107.  2. Patients with following surgical history/medical conditions that may affect drug absorption:  - Gastrointestinal surgery (such as gastrectomy, jejunoileal bypass surgery, small bowel resection)  - Gallbladder surgery (cholecystectomy)  - Innate or acquired serious or uncontrolled gastrointestinal disease  - Coeliac disease  - Bowel obstruction or inflammatory bowel disease (e.g., Crohn’s disease, chronic inflammation)  - Uncontrolled diarrhea (e.g., ≥ Grade 3 diarrhea or < Grade 3 diarrhea which lasts for 7 days or more, even when symptomatic therapy is performed)  - Other inflammatory GI diseases such as graft-versus-host disease of the gut, Bechet's syndrome and scleroderma involving the GI tract  - Any other surgical history/medical conditions that may affect drug absorption at the investigator’s discretion  3. Patients who developed cardiovascular disease (unstable angina, myocardial infarction, stroke, and transient ischemic attack) within 24 weeks prior to study entry, which is deemed to be clinically significant by the investigator.  4. Patients with known active hepatitis B or C infection, or hepatobiliary disease, or known history of immunodeficiency virus infection. However, patients with Gilbert’s syndrome, asymptomatic gallstones, or stable chronic liver disease are, at the discretion of the investigator, eligible for the study. Patients who are hepatitis B carriers may be eligible if they are on antiviral therapy 2 weeks prior to study entry.  5. Patients with neuropathy grade > 2 based on CTCAE v5.0 at the time of study entry.  6. Patients with uncontrolled medical or mental illness that, in the investigator’s judgment, could affect treatment tolerability or compliance.  7. Patients diagnosed with other malignant primary tumor with the exception of the following:  - Malignancy diagnosed at least 5 years previously without evidence of recurrence or  persistent disease  - The complete excision of basal/squamous cell carcinoma or papillary thyroid carcinoma or the complete treatment of cervical intraepithelial neoplasia or other in situ carcinoma.  8. Patients with symptomatic or unstable, untreated metastases to the central nervous system at the time of screening (‘Unstable’ means worsening of symptoms within 4 weeks prior to screening).  9. Patients who are currently receiving alternative cytotoxic agents, regular systemic corticosteroids and medications that could influence drug absorption (e.g., H2-antihistamines, antacids, metoclopramide and charcoal) within 4 weeks prior to entry into the study (C1D1).  10. Patients who are currently receiving (or unable to stop use the day before the first dose of DHP107 and throughout the study) prescription or non-prescription medications or other products known to be moderate or potent inhibitors/inducers of CYP3A4, P-gp, or CYP2C8.  11. Patients who cannot intake whole high-fat meal offered.  12. Pregnant or breastfeeding women.  13. Patients who have received any investigational drugs or devices within 4 weeks before the first day of study treatment (C1D1) |

*C* cycle, *CrCl* creatinine clearance, *CTCAE* Common Terminology Criteria for Adverse Events, *cyp* cytochrome P450, *D* day, *eGFR* glomerular filtration rate, *ULN* upper limit of normal

**Table S2** Validation of the LC−MS/MS method

| Range of calibration curve: | Linear over the range of 0.3 ng/mL - 300 ng/mL |
| --- | --- |
| Lower limit of quantification: | 0.3 ng/mL |
| Selectivity: | No peak with an area higher than 1/5 (20%) than that of analyte and no peak with an area higher than 1/20 (5%) than that of the IS compound of the lowest calibrator sample were detected |
| Intra-day precision of quality control samples: | 1.9% - 8.9% (range: 0.3 ng/mL - 200 ng/mL) for paclitaxel  1.9% - 12.8% (range: 0.3 ng/mL - 200 ng/mL) for 6α-hydroxy-paclitaxel  3.7% - 13.1% (range: 0.3 ng/mL - 200 ng/mL) for 3’-p-hydroxy-paclitaxel |
| Inter-day precision of quality control samples: | 5.0% - 8.2% (range: 0.3 ng/mL - 200 ng/mL) for paclitaxel  6.8% - 8.7% (range: 0.3 ng/mL - 200 ng/mL) for 6α-hydroxy-paclitaxel  7.5% - 9.4% (range: 0.3 ng/mL - 200 ng/mL) for 3’-p-hydroxy-paclitaxel |
| Intra-day accuracy of quality control samples: | -7.7% - 6.7% (range: 0.3 ng/mL - 200 ng/mL) for paclitaxel  0.0% - 11.7% (range: 0.3 ng/mL - 200 ng/mL) for 6α-hydroxy-paclitaxel  -2.3% - 15.7% (range: 0.3 ng/mL - 200 ng/mL) for 3’-p-hydroxy-paclitaxel |
| Inter-day accuracy of quality control samples: | 1.0% - 5.0% (range: 0.3 ng/mL - 200 ng/mL) for paclitaxel  4.7% - 7.3% (range: 0.3 ng/mL - 200 ng/mL) for 6α-hydroxy-paclitaxel  5.7% - 7.3% (range: 0.3 ng/mL - 200 ng/mL) for 3’-p-hydroxy-paclitaxel |
| Matrix effect:  (cv% of is normalized matrix factors) | 7.2% and 12.6% (at 200 ng/mL and 1 ng/mL) for paclitaxel  8.3% and 13.1% (at 200 ng/mL and 1 ng/mL) for 6α-hydroxy-paclitaxel  5.1% and 8.5% (at 200 ng/mL and 1 ng/mL) for 3’-p-hydroxy-paclitaxel |
| Effect of hemolysis with precision: | -2.5% with 2.5% precision (at 200 ng/mL)  2.0% with 6.0% precision (at 1 ng/mL) for paclitaxel  -0.5% with 3.8% precision (at 200 ng/mL)  5.0% with 6.2% precision (at 1 ng/mL) for 6α-hydroxy-paclitaxel  0.5% with 6.1% precision (at 200 ng/mL)  9.0% with 3.3% precision (at 1 ng/mL) for 3’-p-hydroxy-paclitaxel |
| Effect of hyper lipemic plasma with precision: | 0.5% with 5.2% precision (at 200 ng/mL)  -4.5% with 1.0% precision (at 1 ng/mL) for paclitaxel  8.0% with 6.6% precision (at 200 ng/mL)  6.0% with 2.6% precision (at 1 ng/mL) for 6α-hydroxy-paclitaxel  13.0% with 4.9% precision (at 200 ng/mL)  12.0% with 8.1% precision (at 1 ng/mL) for 3’-p-hydroxy-paclitaxel |
| Extraction recovery with precision: | 94.9% with 4.6% precision (at 200 ng/mL)  94.5% with 5.5% precision (at 1 ng/mL) for paclitaxel  92.0% with 2.7% precision (at 200 ng/mL)  95.2% with 7.9% precision (at 1 ng/mL) for 6α-hydroxy-paclitaxel  90.4% with 4.3% precision (at 200 ng/mL)  94.9% with 5.4% precision (at 1 ng/mL) for 3’-p-hydroxy-paclitaxel |
| Extraction recovery of is with precision: | 98.3% with 6.8% precision (at 100 ng/mL) |
| Stability in the processed plasma samples in autosampler: | Stable for all analytes for at least 57 hours stored at 25 °C cover the range of 1 ng/mL - 200 ng/mL |
| Stability for reinjection with calibrators: | Possible within at least 51 hours if the samples are stored at 25°C cover the range of 1 ng/mL - 200 ng/mL |
| Short-term stability in plasma at room temperature: | Stable for all analytes for at least 3 hours at room temperature over the range of 1 ng/mL - 200 ng/mL |
| Freeze/thaw stability: | Stable for all analytes for three cycles over the range of 1 ng/mL - 200 ng/mL |
| Stability in frozen plasma: | Stable for at least 393 days below –70 °C cover the range of 1 ng/mL - 200 ng/mL for paclitaxel and  6α-hydroxy-paclitaxel  Stable for at least 381 days below –70 °C  at 1 ng/mL concentration of 3’-p-hydroxy-paclitaxel  Stable for at least 393 days below –70 °C  at 200 ng/mL concentration of 3’-p-hydroxy-paclitaxel |
| Stability in blood during the sample collection process | Stable for all analytes for at least 60 minutes stored at ambient temperature over the range of 1 ng/mL - 200 ng/mL |
| Dilution effect: | Possible at 5-fold dilution |
| Long run effect: | At least 141 injections can be analyzed within a run |
| Stability in stock and working solutions: | paclitaxel, 6α-hydroxy-paclitaxel, 3’-p-hydroxy-paclitaxel and IS in stock solution (100 µg/mL prepared in methanol): stable for at least 4 hours at room temperature  stable for at least 181 days in a freezer (-30 to -15 °C)  paclitaxel, 6α-hydroxy-paclitaxel and 3’-p-hydroxy- paclitaxel in working solutions (10 ng /mL - 2000 ng/mL), prepared in methanol:  stable for at least 4 hours at room temperature  stable for at least 181 days in a freezer (-30 to -15 °C)  Working solution of IS (1000 ng/mL prepared in methanol: stable for at least 4 hours at room temperature  stable for at least 181 days in a freezer (-30 to -15 °C) |

**Table S3** Safety summary

| Outcome, n (%) | Fasted–fed^a^ (*n*=13) | Fed–fasted^b^ (*n*=12) | All patients (*n*=25) |
| --- | --- | --- | --- |
| AEs | 10 (76.9) | 8 (66.7) | 18 (72.0) |
| Severe AEs | 4 (30.8) | 2 (16.7) | 6 (24.0) |
| Study drug-related AEs | 10 (76.9) | 8 (66.7) | 18 (72.0) |
| Severe study drug-related AEs | 3 (23.1) | 2 (16.7) | 5 (20.0) |
| Serious AEs | 4 (30.8) | 2 (16.7) | 6 (24.0) |
| Serious ADRs | 3 (23.1) | 2 (16.7) | 5 (20.0) |
| AEs leading to permanent discontinuation of DHP107 | 4 (30.8) | 2 (16.7) | 6 (24.0) |

^a^Received DHP107 in fasted state on day 1 and fed state on day 8

^b^Received DHP107 in fed state on day 1 and fasted state on day 8

*ADR* adverse drug reaction, *AE* adverse event

**Table S4** Adverse event summary for the patients who received DHP107 in the fasted and fed states (safety population; n=25)^a^

| **Adverse event, n (%)** | **Fasted** | **Fed** |
| --- | --- | --- |
| Any treatment-emergent adverse event^b^ | 16 (64.0) | 18 (72.0) |
| Serious adverse events^c^ | 0 | 3 (12.0) |
| Treatment-related adverse events | 12 (48.0) | 13 (52.0) |
| Most common treatment-emergent adverse events |  |  |
| Diarrhea | 5 (20.0) | 4 (16.0) |
| Abdominal pain | 1 (4.0) | 2 (8.0) |
| Anemia | 1 (4.0) | 1 (4.0) |
| Vomiting | 2 (8.0) | 1 (4.0) |
| Cough | 2 (8.0) | 0 |
| Decreased appetite | 1 (4.0) | 1 (4.0) |
| Dehydration | 0 | 2 (8.0) |
| Headache | 0 | 2 (8.0) |
| Anxiety | 0 | 1 (4.0) |

^a^Events occurring during the first two weeks of cycle 1 when the fasted and fed states could be clearly differentiated.

^b^Treatment-emergent adverse events were events that started after the first dose of DHP107, or were present prior to the first dose of DHP107 but increased in severity after the first dose.

^c^Dehydration (n=2), and neutropenia (n=1). These three SAEs occurred in two patients and had a fatal outcome in both patients.
